# Examining the Potential Mediating Role of Maternal Mental Health in the Association Between Socioeconomic Deprivation and Child Development Outcomes

**DOI:** 10.1007/s10995-025-04050-5

**Published:** 2025-02-07

**Authors:** Kenneth Okelo, Aja Murray, Josiah King, Iain Hardie, Hildigunnur Anna Hall, Emily Luedecke, Louise Marryat, Lucy Thompson, Helen Minnis, Michael Lombardo, Philip Wilson, Bonnie Auyeung

**Affiliations:** 1 Department of Psychology, School of Philosophy, Psychology and Language Sciences, University of Edinburgh, 7 George Square, Edinburgh, EH8 9JZ UK; 2 Centre for Health Security and Communicable Disease Control, Directorate of Health, Reykjavík, Iceland; 3 School of Health Sciences, University of Dundee, Dundee, UK; 4 Centre for Rural Health, Institute of Applied Health Sciences, University of Aberdeen, Aberdeen, UK; 5 Gillberg Neuropsychiatry Centre, University of Gothenburg, Gothenburg, Sweden; 6 School of Health and Wellbeing, University of Glasgow, Glasgow, UK; 7 Laboratory for Autism and Neurodevelopmental Disorders, Center for Neuroscience and Cognitive Systems, Istituto Italiano di Tecnologia, Rovereto, Italy; 8 Centre for Research and Education in General Practice, University of Copenhagen, Copenhagen, Denmark

**Keywords:** Mental illness, Developmental delays, Infant

## Abstract

**Background:**

Socioeconomic deprivation has been linked to negative child developmental outcomes including brain development, psychological well-being, educational attainment, and social-emotional well-being. Maternal mental health has also been linked to mothers’ parenting practices and their children’s developmental outcomes. However, limited evidence exists regarding the role of maternal mental health (prenatal and postnatal) in the association between socioeconomic deprivation and children’s developmental outcomes.

**Methods:**

We examined the potential role of maternal mental health in the association between socioeconomic deprivation (SED) and child development outcomes. We used a large linked administrative health dataset covering children born between 2011 and 2015 in Greater Glasgow and Clyde, Scotland. Of the 76,483 participants, 55,856 mothers with matched children’s developmental outcome data were included. A mediation analysis model, adjusted for confounders and covariates, was used.

**Results:**

Maternal mental health assessed by a history of hospital admissions mediated, but to a small extent, the relationship between SED and children’s developmental outcomes. The average direct effect (ADE), of SED in the first model with a history of hospital admissions, was ADE: ES = − 0.0875 (95% CI = − 0.097, − 0.08; *p* < 0.001) and ACME: ES = − 0.0002 (95% CI = − 0.001, − 0.0001; *p* = 0.01). The proportion mediated by the history of mental health admission was 0.3%.

**Conclusion:**

The association between SED and children’s developmental outcomes appears to be partially mediated by maternal mental health, although the proportional-mediated effect was very small.

## Significance

Previous research has shown that maternal mental health acts as a mediator in the relationship between a child’s developmental outcomes and various factors, such as maternal childhood adversity, household food insecurity, socioeconomic deprivation, and prenatal stress (Barry et al., 2015a; Lipschutz et al., 2023; Ma et al., 2022; Pedroso et al., 2021; Smith et al., 2023). However, there is limited information available on the role of maternal mental health, both prenatal and postnatal, in the connection between socioeconomic deprivation and children's developmental outcomes.

## Introduction

As of 2022, approximately 4.2 million children were living in low-income households in the UK (Gov.uk, 2023) compared to more than 3.5 billion people globally (WorldBank, 2024). Socioeconomic deprivation has been linked to negative child developmental outcomes, including brain development, psychological well-being, educational attainment, and social-emotional well-being (Barry et al., 2015a; Gibson-Davis et al., 2022; Lee & Zhang, 2022; Rocha et al., 2023; Sosu & Schmidt, 2017). Stronger associations between socioeconomic deprivation and children’s language and cognitive developmental outcomes have been observed compared to other domains, such as physical and psychological well-being (Letourneau et al., 2013). Maternal mental health has been proposed as a potential mediating mechanism; however, limited evidence exists regarding its role in the association between socioeconomic deprivation and children’s developmental outcomes.

Prenatal and postnatal maternal mental health is a risk factor for developmental delays and is associated with offspring mental health outcomes, such as the increased risk of schizophrenia and mood disorders (Goodman et al., 2011; O’Connor et al., 2014). Studies have also suggested that maternal mental health may have long-term developmental problems through elevated hormones that influence the fetal brain’s structure and function (Lupien et al., 2009; Welberg & Seckl, 2001). In addition, there have also been poor obstetric outcomes in women with mental illness, such as birth difficulties, preeclampsia, postpartum depression, preterm delivery, and poor breastfeeding practices (Bowen & Muhajarine, 2006; Bowen et al., 2009; Kingston & Tough, 2014; Talge et al., 2007). Notably, such obstetric outcomes have also been linked to delayed child developmental outcomes (Madlala & Kassier, 2018).

Maternal mental health is also a predictor of parenting practices and child-caregiver attachment and could influence child stimulation (Herba et al., 2016). Notably, positive parenting practices such as good health, adequate nutrition, safety and security, responsive caregiving, and early learning during the first 1000 days (pregnancy to 3 years) promote optimal growth and development (Abboah-Offei et al., 2022; Black, 2019; Olusanya et al., 2023; Wertlieb, 2019). These findings highlight the importance of considering maternal mental health when examining the effects of socioeconomic deprivation on children’s growth and development.

Our study contributes to the body of knowledge by examining the role of maternal mental health, assessed by (a) a history of maternal mental health hospital admissions; (b) psychotropic drug prescription(s) during pregnancy; and (c) psychotropic drug prescription(s) postnatally, in the relationship between socioeconomic deprivation (SED) measured by Scottish Index of Multiple Deprivation (SIMD) and childhood development using a large linked administrative health dataset covering children born between 2011 and 2015 in Greater Glasgow and Clyde, Scotland to inform global health practices. Specifically, we address the following research question:

*RQ1*: Is the association between SED and adverse development reported by mothers to health visitors during offspring early child health reviews mediated by maternal mental health, measured by a history of maternal mental health hospital admissions?

*RQ2*: Is the association between SED and adverse development reported by mothers to health visitors during offspring early child health reviews mediated by maternal mental health, measured by prescriptions related to mental health during pregnancy through postnatal periods (infancy and toddlerhood)?

## Methods

### Setting

This paper reports a secondary analysis of linked administrative health data from parents and children born in Greater Glasgow and Clyde, United Kingdom (UK). According to the Scottish Index of Deprivation (SIMD), 29% of Glasgow’s city population resides in 20% of the most deprived areas in Scotland, while 6% lives in 10% of the least deprived areas in Scotland (Gov.scot, 2022). Children born in Glasgow were twice as likely to have a low birth weight and 50% more likely to be born prematurely (Gov.scot, 2022). Such trends in health indicators have also been observed in socio-economically deprived settings in developed countries (Diguisto et al., 2022; Matković et al., 2007). In addition, Glasgow city as a whole had the lowest mean score on the Warwick-Edinburgh Mental Well-being Scale (WEMWBS), compared to Edinburgh and Aberdeen between 2008 and 2011, with a higher score representing better mental health (Gov.scot, 2022).

### Participants

**Fig. 1:**
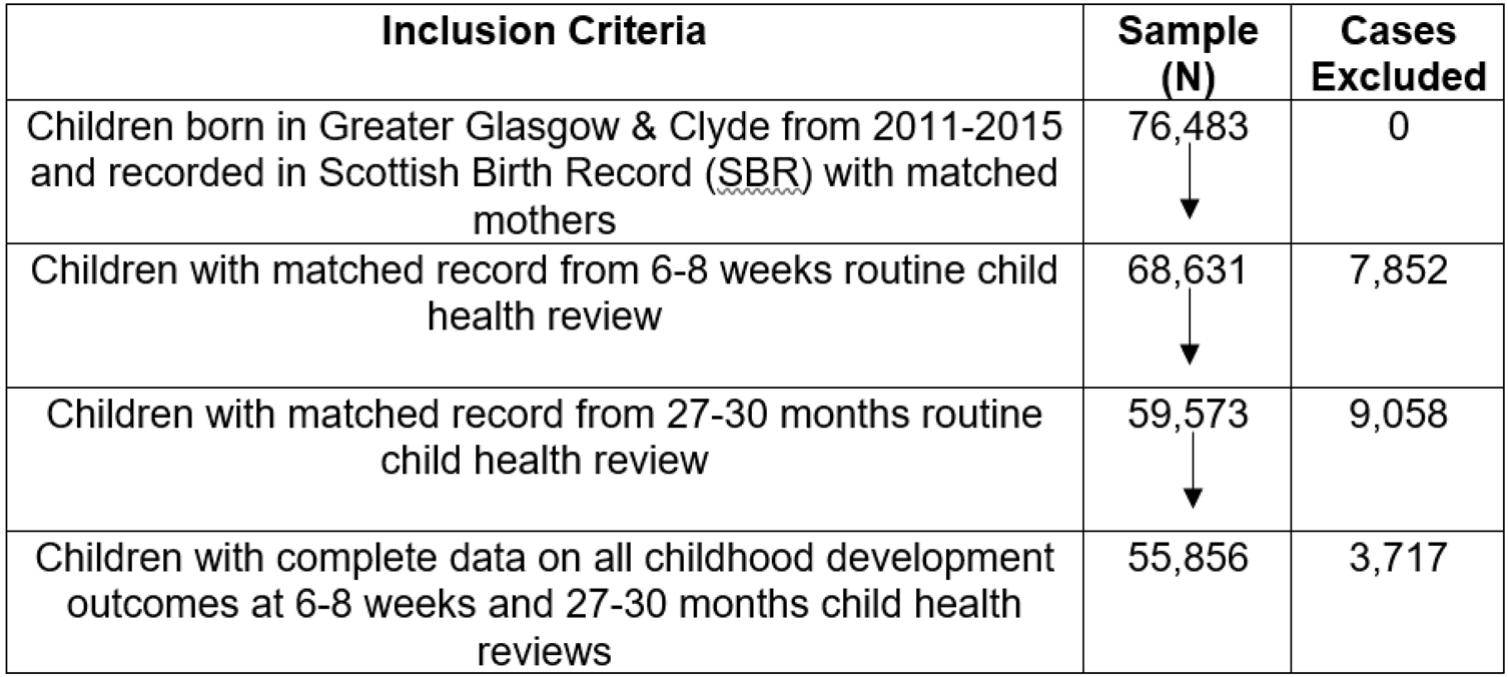
Participants flow chart (inclusion and exclusion criteria)

Participants for this study were drawn from the Child Mental Health in Education (ChiME) project (Barry et al., 2015b). This project Linked administrative health data on all children born in Greater Glasgow and Clyde to map their developmental trajectories from prenatal development through early puberty. We included all children born between 2011 and 2015 with maternal health records and records for universal child health reviews at ages 6–8 weeks and 27–30 months available. The total number of participants in the dataset is 76,483. Only participants with all the variables (child’s review data, mothers’ pregnancy and post-pregnancy data, and SIMD) were included in this study. The final sample size included in this study was n = 55,856 mother–child participants. See Fig. 1.

Overall, the analysis covered 2011–2018 (i.e., the 2011–2015 period in which the children were born, plus data from 2016–2018 to allow all children to undergo their 6–8 weeks and 27–30 months of child health reviews).

### Measures

#### Outcome Variable

**Fig. 2:**
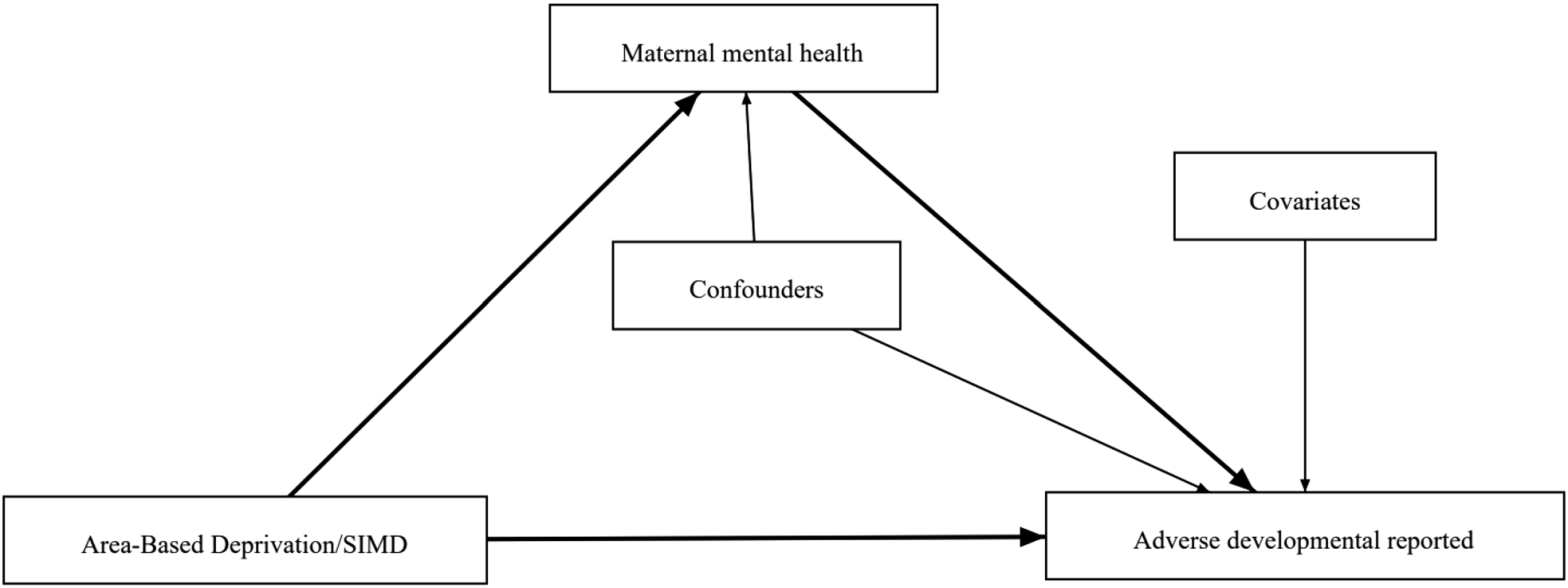
Directed acyclic graph illustrating the hypothesized interaction between variables

The primary outcome variable in our analysis was *adverse developmental concerns r*eported by mothers to health visitors at scheduled health assessments. Adverse developmental concerns/observations were defined as cases, where a concern was raised or observed in at least one developmental domain (personal-social, emotional-behavioural-attention, speech-language, motor skills, vision-social awareness, or hearing-communication). Health visitors make these observations based on the following: (a) elicitation of parental concerns, (b) a structured observation of the child, and/or (c) The Sure Start Language Measure (SSLM) and Strengths & Difficulties Questionnaire (SDQ) (see: Scottish Government, 2015). This is a binary variable indicating whether mothers reported any adverse developmental concern to the health visitors during each child’s 6–8 weeks health review or 27–30 months health review (coded: 0 = no adverse observations made, 1 = adverse observation(s) made). See Fig. 2.

#### Mediator Variables

Our mediator variable was maternal mental health measured by maternal psychotropic drug prescriptions and maternal history of mental health hospital admissions. In our first model, we explored *maternal history of mental health admissions*, recorded during pregnancy as a mediator variable. It was coded as a binary variable (coded 0 = no history of admissions; 1 = history of admissions, i.e. one or more recorded admissions). Data source: Mental health inpatient and day-case hospital admission data. We used hospital records of maternal history of mental health admissions from mental health inpatients and day-case hospital admissions data. This was linked to birth record data from the Scottish Birth Records (SBR) and the National Records of Scotland Births (National Records of Scotland, 2023; see Public Health Scotland, 2022). In the second model, we used ‘*maternal psychotropic drug prescription(s) during pregnancy*’ (first, second, and third trimesters) as a mediator variable. In the third model, we used *maternal psychotropic drug prescription(s) during the postnatal period (birth to 30 months)*. These prescription variables were binary, showing whether mothers received any prescriptions recorded in the Prescribing Information System (PIS) that may have been mental health-related, coded 0 = no record of mental health-related prescriptions, 1 = record of at least one mental health-related prescription. We used data on prescriptions related to mental health from Scotland’s prescription information system (PIS), which is the definitive data source for all medicines prescribed and dispensed in the community (Bennett Institute for Applied Data Science, 2023). We defined mental health-related prescriptions as those included in the British National Formulary (BNF) which are directly related to mental health (Bennett Institute for Applied Data Science, 2023). A full list of prescriptions is provided in Appendix 1. The rationale for using the three measurements for mental health, though proxy measurements, are complementary and give a relatively comprehensive and accurate picture of the prevalence of maternal mental health problems in this study setting. See Fig. 2.

#### Predictor Variable

The predictor variable was SED. These data come from the Scottish Index of Deprivation (SIMD) quintiles and were coded as categorical variables (coded as 1 = least deprived, 2 = less deprived, 3 = medium deprived, 4 = more deprived, and 5 = most deprived). The data source for this was the Scottish Birth Record (recorded at birth and derived from postcode) (Scotland, 2023). See Fig. 2.

#### Confounders and Covariates Variables

Analyses included unadjusted models of the relationship between *mental health* and childhood development outcome variables, as well as models adjusted for the following potential confounders or covariates (i.e. control variables):

##### Confounders

The potential confounders considered in our analysis were *maternal prenatal smoking, maternal infection during pregnancy*, *Maternal infection-related prescription(s) during pregnancy*, *Maternal infection(s) diagnosed in the hospital during pregnancy, marital status, and maternal age*. All of them except maternal age were coded as a binary variable (coded 0 = no, 1 = yes). *Maternal age*; was coded as an ordinal variable (coded: 0 ≤ 19, 2 = 20 – 35, 3 ≥ 36, i.e., in line with the ‘optimal’ (20–35) and ‘suboptimal’ (≤ 19/ ≥ 36) maternal age categorisations for neurodevelopment shown by previous research.

##### Covariates

The child’s sex was considered a covariate in the analysis*. The sex of the child* was coded as a binary variable (0 = male, 1 = female).

### Statistical Analysis

Descriptive statistics Table 1 sets out the characteristics of our study participants, outlining the frequencies of our socioeconomic deprivation variable, maternal mental health variables, and confounders/covariates.

**Table 1:** Descriptive statistics for childhood development outcomes, area-based deprivation, maternal mental health, and confounders/covariates

	N	%	Outcome		Mediator					
			Child developmental outcome—any (i.e. at least one) adverse childhood development outcome		Maternal history of mental health hospital admissions		Psychotropic drug prescriptions during pregnancy		Psychotropic drug prescriptions during postnatal	
			No	Yes	No	Yes	No	Yes	No	Yes
Primary childhood development outcome										
Any (i.e. at least one) adverse childhood development outcome										
No	44,026	78.80%			43,427 (78.4%)	601 (67.8%)	42,412 (79.2%)	1616 (70.0%)	39,694 (79.6%)	4334 (72.2%)
Yes	11,830	21.20%			11,545 (21.6%)	285 (32.2%)	11,139 (20.8%)	691 (30.0%)	10,163 (20.4%)	1667 (27.8%)
SIMD quintile (predictor)										
1 (most deprived)	22,245	39.80%	16,446 (73.9%)	5801 (26.1%)						
2 (more deprived)	10,329	18.50%	8041 (77.8%)	2288 (22.2%)						
3 (medium deprived)	8,452	15.10%	6844 (81.0%)	1608 (19.0%)						
4 (less deprived)	7161	12.80%	6018 (84.0%)	1143(16.0%)						
5 (least deprived)	7669	13.70%	6679 (87.1%)	990 (12.9%)						
Mediator										
Maternal history of mental health hospital admissions										
No	54,970	98.40%								
Yes	886	1.60%								
Psychotropic drug prescriptions during pregnancy										
No	53,551	95.90%								
Yes	2307	4.10%								
Psychotropic drug prescriptions during postnatal										
No	49,857	89.30%								
Yes	6001	10.70%								
Confounders/covariates										
Maternal age at the time of birth										
< 20	2,472	4.40%								
20–35	44,524	79.70%								
> 35	8860	15.90%								
Sex of child										
Male	28,348	50.70%								
Female	27,508	49.30%								
Maternal prenatal smoking										
No	47,908	85.80%								
Yes	7948	14.20%								
Prenatal infections										
Hospital-diagnosed prenatal infection										
No	52,999	94.90%								
Yes	2857	5.10%								
Receipt of infection-related prescription(s) during pregnancy										
No	40,774	73.00%								
Yes	15,082	27.00%								

Further, we conducted a mediation analysis (Xu et al., 2023) to measure the relationship between socioeconomic deprivation, maternal mental health, and childhood development outcomes. In this analysis, the dependent variable (DV) was child developmental outcome, the mediator variable (MV) was maternal mental health, and the independent variable (IV) was SED. We assessed the direct effect of the IV on the DV, known as the Average Direct Effect (ADE). This represents the change in DV when the IV changes, holding the MV constant. The mediation model further assesses the indirect effect of the IV on DV through MV, known as the Average Causal Mediated Effect (ACME). The ACME shows the change in DV due to the change in MV caused by the IV. The total effect, which is the combination of the direct and indirect effect was calculated as the sum of ADE and ACME. The proportional effect represented the proportion of the total effect mediated by the MV.

We used the first model without confounders and covariates and then adjusted the models with the confounders/covariates. In both models, we used the logit function to model the probability of the outcome and the mediator as a function of the predictor and other covariates/confounders. Both covariates and confounders were included in the predictor and mediation model. Given that our outcome and mediator were binary, each participant had two potential values (Y = 1 or 0 and M = 1 or 0), whereas our predictor variable (X) was categorical, and coded as 1 = least deprived, 2 = less deprived, 3 = medium deprived, 4 = more deprived and 5 = most deprived. For the predictor variable, we used 1 (least deprived) as the control value and 5 (most deprived) as the treatment value. We used the mediation function from the mediation R package (Tingley et al., 2014), which uses a Monte Carlo simulation to estimate the mediation effects. That is, it predicts the values of a specific mediator or outcome variable and then computes the average causal mediation, direct effect, and total effects (Valente et al., 2020). Through this, we obtained estimates of the total effects of socioeconomic deprivation on child developmental outcomes, the average direct effect (ADE), and the average causal mediated effects (ACME) for the mediator variable (maternal mental health). In addition, we reported the percentage of the total effect mediated by maternal mental health (de Laat et al., 2018; Smith et al., 2022). All analyses were conducted using the R software within Scotland’s National Safe Haven.

## Results/Findings

### Demographic Characteristics

The population in this study had a relatively high level of deprivation (most deprived at 38.9% and more deprived at 18.5%) relative to Scotland. In terms of mother’s mental health, 1.6% reported having a history of mental health hospital admission, 4.1% had records of psychotropic drug prescriptions during pregnancy and 10.7% had postnatal psychotropic drug prescriptions between 2009 and 2015. Notably, 21.2% of children were reported to have one or more adverse developmental concerns/observations reported by mothers to health visitors during offspring early child health reviews. There were slightly more male children (50.8%) than female children as shown in Table 1.

### Mediation Analysis

The findings suggest the association between SED and children’s developmental outcomes was mediated (but only in a small proportion of the total effect) by maternal mental health, measured by a history of maternal hospital admissions. The findings also show that SED is directly associated with children’s developmental outcomes. In our first model with mental health assessed by a history of admission, the estimates showed a statistically significant direct effect of SED on child developmental outcomes, ADE: ES = − 0.0875 (95% CI [− 0.0969, − 0.08],* p* < 0.001) and a small but a statistically significant indirect effect through the history of mental health admissions (ACME: ES = − 0.0002, 95% CI [− 0.001, − 0.0001],* p* = 0.01). The Total Effect was also significant, ES = -0.097, 95% CI [− 0.0971, − 0.80]). In addition, 0.3% (95% CI = 0.0006, 0.01) of the total effect was mediated by mental health (Table 2). This indicates that a history of mental health admissions mediated the relationship between socioeconomic deprivation and child developmental outcomes.

**Table 2:** Mediation role of maternal mental health (maternal history of mental health hospital admissions) on the association between Socioeconomic deprivation (SIMD -Scottish Index of Deprivation) and child developmental outcomes (adverse developmental observation(s) made by health visitors during early visits)

Mediation model—unadjusted					Mediation model—adjusted				
	Estimate	95% CI lower	95% CI upper	p-value		Estimate	95% CI Lower	95% CI Upper	p-value
ACME^a^	− 0.001	− 0.002	0.001	< 0.001***	ACME	− 0.0002	− 0.0005	− 0.0001	0.01**
ADE^b^	− 0.130	− 0.140	− 0.120	< 0.001***	ADE	− 0.0875	− 0.0969	− 0.080	< 0.001***
Total Effect^c^	− 0.132	− 0.141	− 0.120	< 0.001***	Total Effect	− 0.0878	− 0.0971	− 0.080	< 0.001***
Prop. Mediated^d^	0.010	0.006	0.010	< 0.001***	Prop. Mediated	0.0026	0.0006	0.010	0.01**

Significant at p < 0.01**, p < 0.001***

^a^Average causal mediation effect (ACME)—that is the effects of socioeconomic on child developmental outcomes mediated by maternal mental health

^b^Average direct effect (ADE)—That is the effect of socioeconomic on child developmental outcomes

^c^Total effect—this is the average causal mediation plus the average direct effect

^d^Proportion mediated—this is ACME/Total effects

However, in our second and third models, mental health assessed by psychotropic drug prescription(s) during pregnancy and postnatally did not mediate the association between SED and children’s developmental outcomes. The findings on mental health psychotropic drug prescription(s) during pregnancy show a statically significant direct effect of SIMD on children’s developmental outcomes with ADE: ES = − 0.0878 (95% CI [− 0.0986, − 0.080], *p* < 0.001) with no indirect effect through maternal mental health psychotropic drug prescription(s) (ACME: ES = − 0.0001, 95% CI [− 0.0002, − 0.0001], *p* = 07). The Total Effect, combining both direct and indirect effects, was (ES = − 0.0878, 95% CI [− 0.0.0986, − 0.080]). In addition, 0.07% (95% CI [0.004, 0.010]) of the total effect was mediated by mental health psychotropic drug prescription(s) (Table 3). The findings on mental health, measured by *maternal psychotropic drug prescription(s) during postnatal (birth to 30 months)*, show a statistically significant direct effect of SED on children’s developmental outcomes with ADE: ES = − 0.0874, 95% CI [− 0.0974, − 0.080], *p* < 0.001) and no indirect effect through the mental health (ACME: ES = − 0.0001, 95% CI [− 0.0004, − 0.0003], *p* = *0.5*). The Total Effect, combining both direct and indirect effects, was (ES = − 0.0878, 95% CI [− 0.0975, − 0.080]). In addition, 0.01% (95% CI [0.002, 0.010]) of the total effect was mediated by mental health (Table 4).

**Table 3:** Mediation role of maternal mental health (Psychotropic drug prescriptions during pregnancy) on the association between socioeconomic deprivation (SIMD -Scottish Index of Deprivation) and child developmental outcomes (adverse developmental observation(s) made by health visitors during early visits)

Mediation model—unadjusted					Mediation model—adjusted				
	Estimate	95% CI lower	95% CI upper	p-value		Estimate	95% CI lower	95% CI upper	p-value
ACME^a^	− 0.002	− 0.002	0.000	< 0.001***	ACME	0.0001	− 0.0002	0.0000	0.7
ADE^b^	− 0.130	− 0.140	− 0.120	< 0.001***	ADE	− 0.0878	− 0.0986	− 0.0800	< 0.001***
Total effect^c^	− 0.132	− 0.141	− 0.120	< 0.001***	Total effect	− 0.0878	− 0.0986	− 0.0800	< 0.001***
Prop. mediated^d^	0.014	0.010	0.020	< 0.001***	Prop. mediated	0.0007	− 0.0041	0.0000	0.7

Significant at p < 0.001***

^a^Average causal mediation effect (ACME)—that is the effects of socioeconomic on child developmental outcomes mediated by maternal mental health

^b^Average direct effect (ADE)—That is the effect of socioeconomic on child developmental outcomes

^c^Total effect—this is the average causal mediation plus the average direct effect

^d^Proportion mediated—this is ACME/Total effects

**Table 4:** Mediation role of maternal mental health (Psychotropic drug prescriptions during postnatal) on the association between socioeconomic deprivation (SIMD-Scottish Index of Deprivation) and child developmental outcomes (adverse developmental observation(s) made by health visitors during early visits)

Mediation model—unadjusted					Mediation model—adjusted				
	Estimate	95% CI lower	95% CI upper	p-value		Estimate	95% CI lower	95% CI upper	p-value
ACME^a^	− 0.003	− 0.004	0.000	< 0.001***	ACME	− 0.0001	− 0.0004	− 0.0003	0.5
ADE^b^	− 0.128	− 0.138	− 0.120	< 0.001***	ADE	− 0.0877	− 0.0974	− 0.0800	< 0.001***
Total Effect^c^	− 0.132	− 0.141	− 0.120	< 0.001***	Total Effect	− 0.0878	− 0.0975	− 0.0800	< 0.001***
Prop. Mediated^d^	0.024	0.019	0.030	< 0.001***	Prop. Mediated	0.0011	− 0.0022	0.0100	0.5

Significant at p < 0.001***

^a^Average causal mediation effect (ACME)—that is the effects of socioeconomic on child developmental outcomes mediated by maternal mental health

^b^Average direct effect (ADE)—that is the effect of socioeconomic on child developmental outcomes

^c^Total effect—this is the average causal mediation plus the average direct effect

^d^Proportion mediated—this is ACME/total effects

## Discussion

This study aimed to determine the mediating role of maternal mental health difficulties on the association between SED and children’s developmental outcomes. Results showed a significant association between SED and child developmental outcomes. This supports similar findings in different settings on the association between SED and mental health and children’s developmental outcomes reported in several studies (Marryat et al., 2010; Smith et al., 2023).

The findings also suggest a small but significant mediation effect of maternal mental health, measured by a history of maternal hospital admissions on the association between SED and children’s developmental outcomes. However, this trend was not observed in maternal mental health assessed by psychotropic drug prescription(s). Previous studies have also reported the mediating role of maternal mental health in the relationship between child developmental outcomes and factors such as maternal childhood adversity, household food insecurity, socioeconomic deprivation, and prenatal stress (Barry et al., 2015a; Lipschutz et al., 2023; Ma et al., 2022; Pedroso et al., 2021; Smith et al., 2023). In this context, mothers from the most deprived settings are likely to be worried and stressed about adequate care and provision for their children, which might increase the rates of depression and anxiety among such mothers, thereby affecting their mental health and parenting practices. In addition, children living in such settings are more likely to be exposed to adverse childhood experiences, which in turn could affect the mental health of their mothers (Rowell & Neal-Barnett, 2022).

Mental illness has been linked to disrupted parenting behaviours and insecure child-mother attachment (Goodman et al., 2011). These factors have been associated with poor developmental outcomes (O'Connor et al., 2014). Therefore, this study contributes to existing evidence on the effects of SED and maternal mental health on children’s developmental outcomes. However, the low proportional mediation reported in this study merits further investigation. Future studies are needed to establish other possible mediators of the association between SED and delayed developmental outcomes in children.

The strength of this study is that it uses a very large linked administrative health dataset covering all children born between 2011 and 2015 in Greater Glasgow and Clyde, Scotland, who had data available on maternal health records and early universal child health reviews. Notably, such datasets have large sample sizes thereby reducing sampling error and increasing statistical power (Gavrielov-Yusim & Friger, 2014). In addition, it also offers a large and diverse population, thereby increasing the generalizability of the study findings (Ehrenstein et al., 2017).

However, this study also has the following limitations: since the administrative data obtained were not originally collected for research purposes, they might not be fully representative of the target population because of missing data arising from people not interacting with health services or inaccuracies in data collection and linkage processes (Harron et al., 2017). This can be a particular issue for people facing barriers such as racism or homelessness (Shaw et al., 2022). In the case of our study, coverage of participation in Scotland’s universal child health reviews is known to be lower among those in more deprived areas (Horne et al., 2021), which may result in the participants of our study not being fully representative of all children born in Greater Glasgow and Clyde during our analysis period. Notably, the proxy measurement used in our study for mental health variables and noting that SIMD doesn’t account for individual-based deprivation could also influence our findings. In addition, our study was unable to account for paternal mental health despite its critical role in maternal mental health and children’s developmental outcomes (Kvalevaag et al., 2013). Future studies could incorporate paternal mental health along with maternal mental health as potential mediators in the relationship between socioeconomic deprivation and children’s developmental outcomes.

### Conclusions and Policy Implications

This study aimed to determine the mediating role of maternal mental health on the association between SED and children’s developmental outcomes. The findings showed a statistically significant ACME of maternal mental health measured by a history of maternal hospital admissions on the association between SED and children’s developmental outcomes. However, the proportion mediated was very low. Given the importance of early exposure to children’s holistic growth and development, population-wide studies are needed to generate more fine-grained evidence of the role of maternal mental health in the association between SED and children’s developmental outcomes. In addition, using outpatient data could potentially capture information that might be missed by using our dataset. Further studies are needed to confirm the aspects of SED measured by SIMD that could be responsible for the higher proportion of direct effects on child developmental outcomes.

## Data Availability

Data is not available based on the data access policy of the government agency.

## Appendix 1

*British National Formulary (BNF) chapter 4.1.2*: Anxiolytics; Buspirone hydrochloride**,** Diazepam**,** Lorazepam and Oxazepam.

*BNF chapter 4.2.1*: Antipsychotic drugs; Amisulpride, Aripiprazole, Chlorpromazine hydrochloride, Flupentixol, Haloperidol, Levomepromazine, Olanzapine, Pericyazine, Perphenazine, Chlorpromazine hydrochloride, Promazine hydrochloride, Quetiapine, Risperidone, Sulpiride, Trifluoperazine and Zuclopenthixol.

*BNF chapter 4.2.3*: Drugs used for mania and hypomania: Lithium carbonate and Lithium citrate.

*BNF chapter 4.3.1*: Tricyclic and related Antidepressant drugs; Clomipramine hydrochloride, Dosulepin hydrochloride**,** Doxepin, Imipramine hydrochloride, Lofepramine, Nortriptyline, Trazodone hydrochloride and Trimipramine.

*BNF chapter 4.3.2*: Monoamine-oxidase inhibitors: Moclobemide and Phenelzine.

*BNF chapter 4.3.3*: Selective serotonin reuptake inhibitors; Citalopram, Duloxetine, Escitalopram, Fluoxetine, Paroxetine and Sertraline.

*BNF chapter 4.3.4*: Other antidepressants; Flupentixol, Mirtazapine and Reboxetine.

*Drugs included in BNF Chapter 4*: Central Nervous System which is not directly related to mental health (i.e. drugs used for insomnia, ADHD, obesity, nausea, vertigo, pain/migraines, epilepsy, Parkinsonism, and substance abuse), were not included.

## Abbreviations

SED: Social economic deprivation
SIMD: Scottish index of deprivation
